# Diagnosis of diabetes insipidus observed in Swiss Duroc boars

**DOI:** 10.1186/s12917-016-0645-4

**Published:** 2016-01-29

**Authors:** Alexander Grahofer, Natalie Wiedemar, Corinne Gurtner, Cord Drögemüller, Heiko Nathues

**Affiliations:** Clinic for Swine, Department of Clinical Veterinary Medicine, Vetsuisse Faculty, University of Berne, Bremgartenstrasse 109a, CH-3012 Bern, Switzerland; Institute of Genetics, Department of Clinical Research and Veterinary Public Health, Vetsuisse Faculty, University of Berne, Bremgartenstrasse 109a, CH-3012 Bern, Switzerland; Institute of Animal Pathology, Department of Infectious Diseases and Pathobiology, Vetsuisse Faculty, University of Berne, Länggassstrasse 122, CH-3012 Bern, Switzerland

**Keywords:** Polydipsia, Polyuria, Hyposthenuria, Pig, Antidiuretic hormone, Vasopressin, Water deprivation test

## Abstract

**Background:**

Diabetes insipidus (DI) is a rare disease in humans and animals, which is caused by the lack of production, malfunction or dysfunction of the distal nephron to the antidiuretic effect of the antidiuretic hormone (ADH). Diagnosis requires a thorough medical history, clinical examination and further laboratory confirmation. This case report describes the appearance of DI in five Duroc boars in Switzerland.

**Case presentation:**

Two purebred intact Duroc boars at the age of 8 months and 1.5 years, respectively, with a history of polyuric and polydipsic symptoms had been referred to the Swine Clinic in Berne. Based on the case history, the results of clinical examination and the analysis of blood and urine, a tentative diagnosis of DI was concluded. Finally, the diagnosis was confirmed by findings from a modified water deprivation test, macroscopic examinations and histopathology. Following the diagnosis, three genes known to be involved in inherited DI in humans were analyzed in order to explore a possible genetic background of the affected boars.

**Conclusion:**

The etiology of DI in pigs is supposed to be the same as in humans, although this disease has never been described in pigs before. Thus, although occurring only on rare occasions, DI should be considered as a differential diagnosis in pigs with polyuria and polydipsia. It seems that a modified water deprivation test may be a helpful tool for confirming a diagnosis in pigs. Since hereditary forms of DI have been described in humans, the occurrence of DI in pigs should be considered in breeding programs although we were not able to identify a disease associated mutation.

## Background

Primary disorders of water balance, such as diabetes insipidus (DI) or psychogenic polydipsia, belong to the ‘polyuric and polydipsic complex’. DI is characterized by polyuria, a markedly decreased urine specific gravity and compensatory polydipsia without other findings. Combining a thorough clinical examination with specific laboratory testing for particular causes (e.g., diabetes mellitus, hyperthyroidism, pyelonephritis, chronic renal failure), the diagnosis can be established and several other differential diagnoses can be ruled out [1, 2]. In order to verify a presumptive diagnosis, a water deprivation test, an antidiuretic hormone (ADH) stimulation test as well as measurement of endogenous ADH have been described [1–4].

DI is caused by an inadequate secretion, release or activity of ADH [5–8]. The ADH, also known as vasopressin, is a neurohypophyseal peptide hormone and its most important function, maintained through interaction with the V2-receptor in the kidney, is to increase water reabsorption [9]. Any distinction of the several forms of DI cannot solely relay on clinical examination, because symptoms are rather unspecific. A central DI (CDI) occurs due the inadequate secretion of ADH, whereas nephrogenic DI (NDI) is characterized by an insufficient or absent response of the distal nephron to the antidiuretic effect of vasopressin [6–8, 10]. The gestational DI (GDI) is caused by the enzyme ‘cysteine aminopeptidase’, which is produced in the placenta and degrades ADH [6–8].

DI has rarely been reported in pets and farm animals [10, 11]. In humans the disease occurs with a prevalence of 1:25,000 [8]. From these cases less than 10 % can be attributed to a hereditary background. In this context, three different genes are often analyzed in order to identify the form of DI [14]: the arginine vasopressin gene (*AVP*), the arginine vasopressin receptor 2 gene (*AVPR2*) and the vasopressin-sensitive water channel gene (*AQP2*).

In most of the human CDI cases the disease is acquired and can have various underlying etiologies, such as tumors, trauma, infections or central nervous system malformations [12, 13]. There are also familial forms caused by mutations in *AVP*. Most of these mutations are inherited in a dominant manner [14] though recessive cases have been reported [15]. The time of onset of first clinical symptoms in familial CDI shows huge variation and it usually occurs after one year of age [14]. The acquired forms of NDI are often secondary diseases following other metabolic disorders (e.g. diabetes mellitus), urinary tract diseases or drug abuse [12]. Familial NDI can either be caused by mutations in the *AVPR2* or *AQP2* gene [10, 14]. In both circumstances, symptoms usually manifest themselves in the first weeks after birth [14]. Two particular *AVPR2* mutations [14] are responsible for about 90 % of familial NDI cases [10], they cause an X-linked recessive form of NDI and therefore mainly affect male individuals [14] with a frequency of 4–8 per 1 million man born alive [16]. Furthermore, there are autosomal dominant and recessive variants of NDI [17]. Mutations in *AQP2* are the least frequent cause of familial DI. In most of the cases it is inherited in an autosomal recessive way [10].

This case report describes the findings in Duroc boars with DI. The clinical symptoms were partly in accordance with case reports in other animal species. To the authors’ knowledge, this is the first report of the DI in pigs and specifically in Duroc boars.

## Case history

Two purebred Duroc boars were referred to the Swine Clinic Berne, Vetsuisse-Faculty, for further investigation of polyuric and polydipsic symptoms combined with reduced growth despite maintenance of appetite. Owners of the Duroc boars were informed about the possible examinations in written form and they agreed upon Terms of Services that include the intention to publish descriptions of clinical cases in reports. Both animals had a significant drop of semen quality and were excluded from the semen collection process in a boar stud. The first symptoms appeared around a month before referral to the clinic and no other relevant medical history was reported. No treatment was administered by the herd attending veterinarian or animal care taker prior to presentation of the case in the clinic. The boars were kept in individual pens interspersed with straw. In the boar stud, the animals were fed with commercial feed and were provided with fresh water from the public supplier. Water was freely available through a nipple drinker system. The vaccination program included an immunization against *Erysipelothrix rhusiopathiae* twice and *porcine parvovirus* once a year. A yearly deworming of all boars was not conducted, because of negative results from regular examinations of faeces. According to the EU-regulations the boar stud was also tested for Classical swine fever, *Brucella spp.* and *Foot-and-mouth disease virus* and in addition for *Porcine reproductive and respiratory syndrome virus*, *Porcine herpesvirus 1* and *African swine fever virus*. During the interview with the herd attending veterinarian and the thorough clinical history three very similar cases were further identified, where some years ago Duroc boars had shown identical signs. In these three cases the herd attending veterinarian had performed on-farm necropsies. Furthermore, samples from each boar had been sent to a laboratory for diagnostic purpose including the histopathology and bacteriological investigation of the urinary tract system. Also a physical-chemical urinalysis was conducted. At necropsy and the histopathological examination no distinctive gross lesions were found that could explain the polyuric and polydipsic disorders. Apart from this, pathogens not specific for urinary tract infection were found with low quantity in culture, likely due to environmental contamination. All of the urine samples from these three pigs showed a highly decreased specific gravity without other abnormalities.

### Case #1

Case #1 was a 1.5 year old intact Duroc boar with a body weight of 172 kg. Clinical examination revealed the boar was alert and in a moderate body condition. The boar showed a dull, ruffled bristled coat and the integument had multiple superficial skin wounds. The boar had several lesions with an exudative inflammatory process on the ears as well as lateral to the left carpal joint and lateral to the right tarsus. The rectal body temperature was 38.6 °C. The heart rate was 116 beats per minute and the heart sound was slightly muffled. The respiratory rate was 16 breaths per minute, with the animal showing a costoabdominal, abdominal breathing type and a moderate expiratory as well as a slight inspiratory respiratory noise. Based on the examination of the mucous membrane there was currently no evidence that the animal had a circulatory insufficiency. The neurological examination revealed no pathological findings.

During abdominal ultrasonography, the cranial part of the urinary bladder could not be identified, because the *vesica urinaria* extended below the ribs. The bladder was completely distended with echogenic urine. No sediment was observed in the urine. The thickness, the regularity of the bladder wall and mucosal relief, all dependent on the bladder’s volume, i.e. filling, were evaluated and assessed as being physiological. During the clinical examination no urination could be observed.

The genitals were evaluated visually, by digital palpation and by ultrasound examination. The two testes were located in and freely moveable within the scrotum. The scrotum had several skin abrasions and a hard thickening of the skin around 10×4cm on the left lateral side. There was a physiological asymmetry of the testes with both being around 1.5 fists big, but one being slightly bigger than the other one. The tissue was soft and elastic and no pain reaction could be recognized by palpation. No abnormality was found during the ultrasound scan.

Results from the concurrent blood examination and the urine analysis are listed in Tables 1 and 2.

**Table 1:** Blood parameters of case #1

Parameter	Unit	Patient’s value	Reference value
Hematocrite	l/L	0.33	0.33–0.45
Erythrocytes	10e12/L	6.24	6.3–8.8
Leukocytes	10e9/L	**18.74**	7.9–18.5
Sodium	mmol/L	127	129–150
Potassium	mmol/L	4.54	4.26–6.99
Chloride	mmol/L	**87**	96–110
Calcium	mmol/L	2.35	2.32–2.92
Phosphorus	mmol/L	**1.59**	2.06–3.25
Magnesium	mmol/L	**0.75**	0.86–1.21
Iron	μmol/L	17.2	9.5–29.1
Glucose	mmol/L	6.01	4.0–6.6
Total protein	g/L	66.4	54–83
Albumin	g/L	36.6	27.3–39.5
Urea	mmol/L	5.47	3.15–8.07
Creatinine	μmol/L	95	39–130
Bilirubin	μmol/L	1.1	1.0–6.4
ALAT (GPT)	IU	102	41–160
ALP	IU	20	50–303
ASAT (GOT)	IU	37 °	30–139
CK	IU	1020	0–2687
GLDH	IU	**1** °	2–11
LDH	IU	1172	909–2172
SQR	IU	0 °	0–8

°possible interference due to haemolysis

Values not within the physiological range are marked in bold

**Table 2:** Urine parameters of case #1

Parameter	Patient’s value
Colour	diluted
Transparency	clear
Specific weight	1002
Nitrite	negativ
pH-Value	6.0
Protein	negativ
Glucose	negativ
Ketone bodies	negativ
Urobilinogen	negativ
Bilirubin	negativ
Blood	negativ
Squamous epithelium	(+)

Macroscopic examination and histopathology were performed and are described in the section ‘gross examination and histopathological findings’.

### Case #2

Case #2 was an 8 months old intact Duroc boar with a body weight of 133 kg. The clinical examination revealed the boar was alert and in a moderate body condition. The bristles and integument were in a good condition, although there were multiple marble sized, firm nodules on both ears and decubitus ulcerations on both carpi and the left tarsus. The rectal body temperature was 37.8 °C. The heart rate was 100 beats per minute and the heart sound was slightly muffled. The respiratory rate was 20 breaths per minute, with the animal showing a costoabdominal, abdominal breathing type and a moderate expiratory respiratory noise. Based on the examination of the mucous membrane there was currently no evidence that the animal had a circulatory insufficiency. The animal showed a slightly arched back and tripling in hindquarters. The neurological examination revealed no pathological findings, except of proprioceptive deficits in hindquarters. Moreover, the panniculus reflex was slightly decreased from pelvis until thorax and thereafter moderately increased.

During abdominal ultrasonography, the cranial part of the urinary bladder could not be identified, because the *vesica urinaria* extended into the ribcage. The bladder was completely distended with echogenic urine and no sediment was observed in the urine. The thickness, regularity of the bladder wall and mucosal relief, all dependent on bladder’s volume, were evaluated and assessed as being physiological. During the clinical examination urination could be observed nearly every half hour.

The genitals were evaluated visually, by digital palpation and by ultrasound examination. Both testes were located and freely moveable within the scrotum. There was a physiological asymmetry of the two fist-sized testes. The tissue was soft and elastic and no pain reaction could be recognized by palpation. No abnormality was found during the ultrasound examination. The caudal part of the paired bulbourethral gland was manually palpated. The consistency and the size of the gland revealed no pathological findings.

A blood sample was taken and a complete blood count was performed. Furthermore, the concentration of Thyroxin (T4) was determined in order to exclude hyperthyroidism. The results are listed in Table 3. For further diagnostic examinations and as a support for the final diagnosis an ‘abrupt water deprivation test’ was conducted. The body weight was measured and a urine sample was taken and examined immediately prior to the trial (Table 4). Then the animal was completely deprived of water and food for 6 h. During the test, the general condition was monitored every 30 min. After the water deprivation test a 6.7 % loss of body weight was assessed and the urine specific gravity was marginally increased from 1.001 to 1.008.

**Table 3:** Blood parameter of case #2 including Thyroxin (T4)

Parameter	Unit	Patient’s value	Reference value
Hematocrite	l/L	0.33	0.33–0.45
Erythrocytes	10e12/L	6.60	6.3–8.8
Leukocytes	10e9/L	**20.98**	7.9–18.5
Sodium	mmol/L	140	129–150
Potassium	mmol/L	4.53	4.26–6.99
Chloride	mmolL	100	96–110
Calcium	mmol/L	2.44	2.32–2.92
Phosphorus	mmol/L	**1.93**	2.06–3.25
Magnesium	mmol/L	**0.76**	0.86–1.21
Iron	μmol/L	17.5	9.5–29.1
Glucose	mmol/L	**7.37**	4.0–6.6
Total protein	g/L	68.3	54–83
Albumin	g/L	37.1	27.3–39.5
Urea	mmol/L	3.88	3.15–8.07
Creatinine	μmol/L	79	39–130
Bilirubin	μmol/L	**0.8**	1.0–6.4
ALAT (GPT)	IU	75	41–160
ALP	IU	**34**	50–303
ASAT (GOT)	IU	46 °	30–139
CK	IU	278	0–2687
GLDH	IU	2 °	2–11
LDH	IU	**900** °	909–2172
SQR	IU	**1** °	0–8
Thyroxin (T4)	ug/L	44	42–66 *

°possible interference due to haemolysis

*reference range from Anderson et al. [36]

Values not within the physiological range are marked in bold

**Table 4:** Comparing urine parameter before and after the water deprivation test

Parameter	*Before water deprivation test*	*After water deprivation test*
Body weight (kg)	133	124
Colour	diluted	bright yellow
Transparency	clear	turbid
Specific weight	1.001	1.008
Nitrite	negativ	negativ
pH-Value	5.8	6.1
Protein	negativ	negativ
Glucose	negativ	negativ
Ketone bodies	negativ	negativ
Urobilinogen	negativ	negativ
Bilirubin	negativ	negativ
Blood	0-1	negativ
Squamous epithelium	+	+++
Round cell	(+)	+
Amorphic crystal	-	+++

**Fig. 1:**
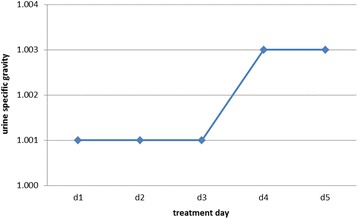
Line diagram displaying the development of the urine specific gravity over treatment time. Data were obtained during desmopressin administration with a Duroc boar suffering from polydipsia and polyuria

Based on the results of the abrupt water deprivation test it was decided to try a therapy with 3-5 drops of Desmopressinacetat (Minirin® solution for intranasal application) into the conjunctival sac every 8 h for 5 days. During the treatment period a clinical examination and measurement of the urine specific gravity was conducted daily. Just a slightly increase of the urine specific gravity was observed (Fig. 1). Five days after the last treatment with Desmopressinacetat, a blood sample was taken and analyzed for Copeptin with an immunoassay for humans. The concentration of Copeptin was lower than 0.8 pmol/l in the serum.

For further diagnostics a necropsy was performed and completed by histopathological examinations. The results are described in the section below.

## Gross examination

### Case #1

The boar showed multiple skin abrasions on various parts of the body. The soles of all claws showed fissures.

Bilateral the cranial lung lobes were affected by a chronic suppurative bronchopneumonia and about 20 % of the lung tissue was affected. The pericardium contained 0.1 l (L) of clear, serous fluid.

**Fig. 2:**
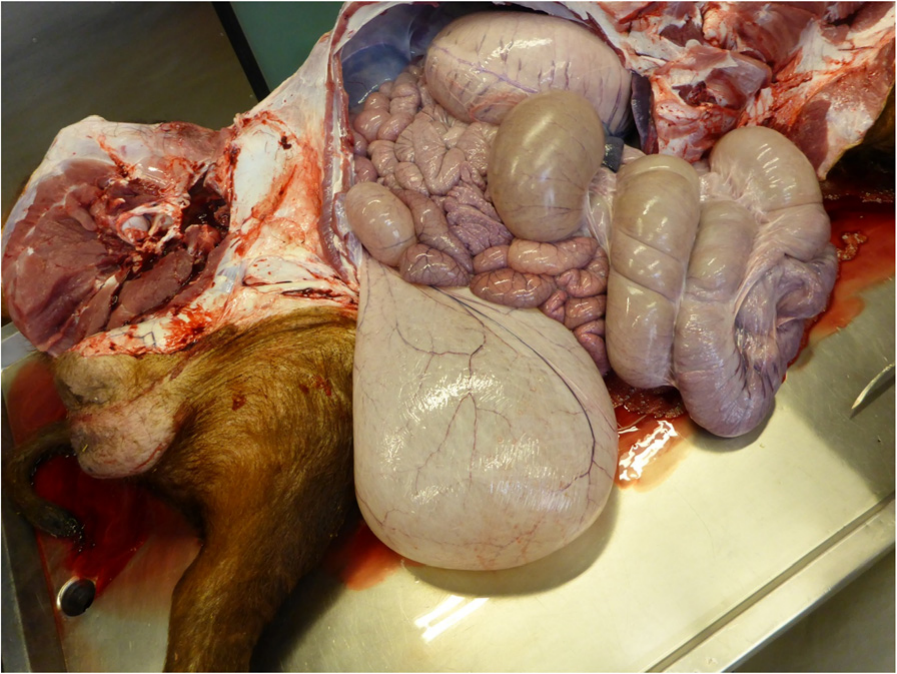
Macroscopic examination of a Duroc boar suffering from polydipsia and polyuria: enlarged urinary bladder

The urinary bladder contained approximately 9 L of clear yellowish urine (Fig. 2). The mucosal lining of the urinary bladder was intact and the lumen of the urethra was free from obstruction. The testes were softer than normal on palpation.

### Case #2

Like the first animal, the boar showed multiple skin abrasions and the claws showed fissures. The boar also suffered from a bilateral chronic suppurative bronchopneumonia which affected about 20 % of the lung tissue. The pericard contained about 1 L of serous and clear fluid. The boar had multiple renal cysts in both kidneys of up to 0.5 cm in diameter. The urinary bladder was filled with 3 L of clear yellowish urine and the mucosa was without changes. The lumen of the urethra was free. Both testes were softer than normal on palpation.

## Histological findings for both boars

The macroscopically affected parts of the lung showed a severe infiltration with degenerated neutrophils into the lumen of bronchi and bronchioli and extending into the alveoli. Additionally, there was exudation of fibrin admixed with necrotic debris and proliferation of fibroblasts.

In both pigs the tubuli seminiferi were mostly devoid of mature spermatids and contained a reduced amount of spermatogonia. Additionally, there was also a multifocal reduced amount of sertoli cells. The interstitium of the testes contained few foci of lymphocytes with a mild amount of edema.

In the gray and white matter of the spinal cord of case #1, there were multiple swollen and hypereosinophilic axons (spheroids). Additionally, in the white substance along the whole length of the spinal cord there were few glial nodules.

In the interstitium of the kidneys of boar 1, there were few foci composed of lymphocytes and a lesser amount of macrophages. Some renal tubuli of case #1 contained a small amount of eosinophilic proteinaceous material. Few tubuli of case #2 showed mild multifocal degeneration of epithelial cells.

The corpus of the urinary bladder showed no histological changes.

Multifocal in the hypophysis there were small amounts of mineralized colloid. No special findings were present in the hypothalamus.

## Bacteriological investigation

A bacteriological examination of the affected lung from case #1 yielded a moderate to high concentration of *Trueperella pyogenes* and *Pasteurella multocida subsp. multocida*.

## Genetic analysis

**Fig. 3:**
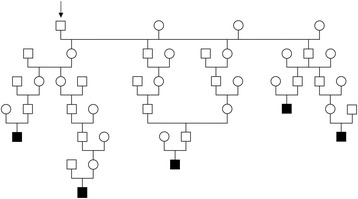
Pedigree of five Duroc boars affected by polydipsia and polyuria. All the cases (shown in black) can be traced back to a particular number of common ancestors. The closest common ancestor is shown in the figure (*labelled with an arrow*). It’s a boar which is 3, 4, 4, 5 and 6 generations away respectively

With the aim of examining a possible genetic background of the disease, the pedigrees of the five affected boars were analyzed. All cases can be traced back to a total of 101 common ancestors, the closest of them 3, to 6 generations away from the affected animals (Fig. 3). According to the knowledge about the genetic causes of DI in humans, the annotated exons of the three functional candidate genes *AVP*, *AVPR2* and *AQP2* were sequenced in material obtained from the affected boars and from healthy animals serving as controls, which have been collected in our laboratory in the course of other ongoing studies. The DNA was isolated either from EDTA-blood using the Nucleon Bacc2 kit (GE Healthcare) or from ear punch biopsies using QIAGEN’s DNeasy spin kit according to the manufacturers’ instructions. Exon-spanning primers (Table 5) were designed using Primer3 software [17] after masking of repetitive sequences with RepeatMasker [18]. PCR reactions were carried out in 10 μl volumes with 5 pmol primer, 5 μl Amlitaq Gold 360 Master Mix (LifeTechnologies), 1 μl 360 GC Enhancer (LifeTechnologies) and ~20 ng of genomic DNA. PCR-products were amplified using GeneAmp 9700 thermocycler (LifeTechnologies), the amplification conditions were 10 min at 95 °C, 32 cycles of 30 s denaturation at 95 °C, 30 s annealing at 60 °C and 1 min elongation at 72 °C, followed by a 7 min hold at 72 °C. To remove redundant primers and nucleotides PCR-products were purified with 1 unit exonuclease I (Roche) and 0.5 units of shrimp alkaline phosphatase (New England BioLabs) in a 30 min incubation step at 37 °C, followed by a 15 min inactivation step at 80 °C. Subsequently the PCR-products were sequenced with the BigDye Terminator v.3.1 cycle sequencing kit (LifeTechnologies) at the following conditions: a hold of 96 °C, followed by 25 cycles of 10 s at 96 °C, 5 s at 50 °C and 2 min at 60 °C. Sequencing-products were resolved on an ABI 3730 capillary sequencer (LifeTechnologies) and the obtained sequence data was analyzed with Sequencher 5.1 software. The sequences were compared to the pig reference sequence and deviations from the reference (variants) were searched for in the affected animals (Table 6). As the mode of inheritance is not defined, both homozygous and heterozygous variants were considered. Subsequently, variants, which were present in the healthy controls, were excluded. In *AVP* three exonic single nucleotide polymorphisms (SNPs) were found in the cases, but all of them were also present in homozygous state in control animals. In *AVPR2* two exonic SNPs were found in four of the cases but all of them were present in healthy control animals as well. In *AQP2* no variants were found in the cases.

**Table 5:** Primer used for sequencing of DNA from Duroc boars affected by polydipsia and polyuria and from negative controls

Gene	Chr	Position exons	Exon	Forward primer	Reverse primer	Length
*AVP*	17	36950915-36951078	1	CATCTTGACCACACCACTGC	TGTCCTAGTCCACCCGACA	576 bp
		36952271-36952472	2	CCGAGCGAATCAGTAGCTTT	TCGGTCACGCAGCTCTCT	546 bp
		36952626-36952826	3	GCTGCCAGGAGGAGAACTAC	ATCGCTTCCCCTACAGGATT	628 bp
*AVPR2*	X	142203295-142203330	1	CAGGAAGGGAAGCAGAGGA	CTACGCTCCTCTCGGGACT	390 bp
		142203810-142203859	2	CAAACCTGGTCAGGCTAAGG	CTGCCAGCAGAACACACATC	496 bp
		142204155-142205039	3a	GCCTCTCCCCAGTAAGATGA	GCTGAGAAGCAGCGAGAAG	747 bp
			3b	GTGGCTCTGTTCCAAGTGCT	CGTAGATCCAGGGGTTGGTA	848 bp
		142205173-142205695	4	GAAGGTGGGTGTGGCTGT	ACTGCTCAAGGCCAGCTC	848 bp
*AQP2*	5	16095902-16095541	1	AACTCCACCTCCAACTCACG	ATGTGTCTGGCTCCAGCATT	683 bp
		16092242-16092078	2	CAGGAAGAAGGCATCCGTAG	CCCCAGGAGGAGGACTGT	828 bp
		16091774-16091694	3			
		16091033-16090821	4	TTACATGGATGCGCCTTTG	CTCAGGGCAGGGGATCTT	497 bp

**Table 6:** Sequence variants detected in two candidate genes from Duroc boars suffering from polydipsia and polyuria

Gene	Chr	Position	dbSNP NCBI_ss#	Genotypes cases			Genotypes controls		
				ref/ref	ref/var	var/var	ref/ref	ref/var	var/var
*AVP*	17	36952357A > G	1966414018			2			1
		36952471 T > C	1966414019		3	2		1	1
		36952785 T > C	1966414020		3	2		1	1
*AVPR2*	X	142204712A > C	1966414021	1		4			2
		142205642C > T	1966414022	1		4	9		11

The positions of the variants are given based on the current assembly (SGSC Sscrofa10.2/susScr3). All the variants are single nucleotide polymorphisms (SNP) whereas the first letter represents the reference nucleotide and the second letter represents the variant (e.g. A > G means a nucleotide exchange from an adenine to a guanine). The SNPs were submitted to dbSNP (www.ncbi.nlm.nih.gov/SNP/) and the submitted SNP (ss) number is reported and will be publicly available with the dbSNP Build 147 release. The genotypes of the cases are displayed separately from the genotypes of the control animals, whereas the number of animals carrying a specific genotype is given. Ref/ref means homozygous for the reference allele, ref/var heterozygous and var/var homozygous for the variant/SNP

## Discussion

Clinical signs, findings of the physical examination and results of further diagnostic methods confirmed the diagnosis of DI in two Duroc boars originating from a semen collection centre. There are several forms of DI described, but in the present study it was not possible to classify the type of DI accurately, as there are some limitations in diagnostics for pigs.

With a clinical history characterized by polyuria and polydipsia in pigs several differential diagnoses have to be kept in mind and each one has to be excluded from the list of differential diagnosis. Therefore, clinical examination and further tests are essential and need to be performed sequentially. During the physical examination the urinary bladder of both boars were extremely full and distended, but dehydration, which is often reported in DI [19], could not be observed. It is noteworthy that in pigs there is no adequate method to measure the hydration status and therefore experimental studies in DI use diuretic medication [20] or fluid restricted pigs [21] for evaluation. As a next step, analysis of the urine was performed and revealed a hyposthenuria with a specific gravity of 1.001 in one boar and 1.002 in the other boar. The specific gravity in pigs, which is the lowest among animals, usually is 1.020 on average and can range between 1.010 and 1.050 [22, 23]. No further abnormalities such as glucosuria or inflammatory signs were detected. Several blood compounds are able to provoke dysfunction of the renal system, hence a complete blood count was conducted and showed a slight chronic inflammation, which might have been caused by the pneumonia.

As a further diagnostic approach a modified water deprivation test was performed. The test is designed to determine, whether ADH is released in response to dehydration and if the kidneys are able to respond normally to the hormone [1–3]. The aim of the test is to achieve maximal ADH secretion and thereby higher concentrated urine. This commonly occurs after a 3 to 5 % loss of body weight due to water loss, which makes it necessary to measure the body weight several times. Additionally, emptying of the bladder with a catheter is required [1]. Due to the characteristic anatomy of the urethra of male pigs, transurethral catheterization of the bladder is impossible [24]. The endpoint of the water deprivation test is a loss of body weight greater than 5 % or a specific gravity of the urine higher than 1.030 [1]. A more accurate measurement is the assessment of the urine’s osmolality [1]. Unfortunately, this was not feasible throughout the whole water deprivation test, because of logistical and financial reasons. The osmolality of urine can also be approximated by multiplying the last two digits of the urine specific gravity by 36 [25]. There was one single measurement of urine’s osmolality of case #2, which was performed during the trial and revealed a result of 32.5 mOsm/Kg. Comparing the measured value with the calculated urine osmolality of 36 mOsm/Kg, there was a good correlation, which can also be observed in other species, e.g. in dogs.

The pig is a good model for renal research. The ratio between urine and serum osmolality in healthy pigs and healthy humans is 3.3 [21]. In the literature, there are numerous different equations described to calculate serum osmolarity in humans. One group reported an equation (Eq. 1) derived from results of an experiment in pigs, where concentrations are expressed in mEq/L [26].

Equation 1:

$$ \mathrm{Serum}\ \mathrm{osmolarity}\ \left(\mathrm{mEq}/\mathrm{l}\right) = 1.8177\ *\ \left[\mathrm{N}\mathrm{a}\right]\ \left(\mathrm{mEq}/\mathrm{L}\right) + \left[\mathrm{Urea}\right]\ \left(\mathrm{mEq}/\mathrm{L}\right) + \left[\mathrm{Glucose}\right]\ \left(\mathrm{mEq}/\mathrm{L}\right) + 26.05 $$

A baseline of 294.9 with a SD of ± 1.8 mOsm/Kg in 10–40 kg femal Yorkshire-Duroc crossbred pigs was reported [27]. Furthermore, in fattening pigs 284.74 ± 5.73 mOsm/Kg [26] was described. In order to get an indication for serum osmolarity in boars, additional three Duroc boars at the age of 8 month to 1.1 year from the semen collecting centre were tested. A reference range from 321 to 326 was measured. The calculated value of serum osmolarity in case #2 was 290.9 mOsm/Kg and, thus, within the above mentioned reference values taken from the literature. In contrast, the calculated value was significantly lower than those measured in the control boars. However, the result generally must be interpreted with caution, because there is a significant bias by food and water intake [28], which can have an impact on the level of osmolarity. If we compare the ratio of serum osmolarity with urine osmolality it is almost three times higher than the average value of healthy pigs.

After the water restriction test a therapeutic attempt was tried, as it is commonly performed with small animals and also with horses. The animals are treated with desmopressin acetate (DDAVP), a synthetic analogue of ADH. There are several routes of administration available, but most often the conjunctival route is chosen. Therefore, one to two drops are applied into the conjunctival sac of both eyes every 12 to 24 h [3, 29]. The eye drop method is a non-invasive, practical and effective way of hormone administration [30]. An oral application of desmopressin is possible, but the bioavailability is lower compared to the afore mentioned method [19, 31]. The duration of the effect of DDAVP varies from eight to 24 h [3]. Since no specific dosage regime was available for pigs, a treatment of the boar 2-3 times per day with 3–5 drops in the conjunctival sac of one eye was proposed. Only a slight decrease of the urine specific gravity was observed. In small animals this result would lead to diagnosis of primary NDI. However, the porcine vasopressin contains a lysine residue in position 8 (lysine-vasopressin), which makes the pig quite different from other mammalian species, where vasopressin contains an arginine residue in position 8 (arginine-vasopressin) [9]. Therefore the porcine V2 receptor has less sensitivity to desmopressin than human V2 receptors. In the literature a two hundred times lower affinity of desmopressin on porcine V2 receptor is described [32]. Even in high doses desmopressin did not induce any hematological response [33]. The authors clinical interpretation is that desmopressin cannot be recommended for diagnostics or treatment in pigs due to the implications mentioned above.

Unfortunately, we performed both diagnostic approaches, therapeutic attempt and modified water deprivation test, only in one boar, because it was only possible to keep one boar in our facilities, regarding to Swiss legislation. To prove the efficiency for both tests in a larger number of pigs with DI, warrants further investigation.

Another diagnostic tool for the confirmation of the disease’s aetiology is the measurement of endogenous ADH in plasma, where in case of diseases osmotic and cardiovascular homeostasis are disturbed [4]. However, the reliability of assessments of plasma ADH levels is poor because the hormone is unstable, largely attached to platelets, and rapidly cleared from plasma [34, 35]. Therefore, the level of a precursor of ADH, Copeptin, which is stable for days, is usually measured in plasma samples [35]. Test kits, specifically designed for pigs, are available for the purpose of clinical research, but they are rarely used and in the presented case the authors could not find a laboratory offering the determination of Copeptin in swine plasma.

The genetic analysis revealed that all the candidate variants in the three DI candidate genes were obviously not associated with the disease and therefore no genetic explanation of the phenotype was found by sequencing of these three genes. Pedigree analysis showed a large number of common ancestors among the affected cases indicating inbreeding. But these shared ancestors are some generations ago which decreases the likelihood of a common inherited simple recessive mutation. Nevertheless, a dominantly inherited mutation with a late onset of clinical signs could also be the cause of the disease, as for example human CDI is caused by dominant mutations of *AVP* [14]. A recessive inheritance analogue to *AQP2* mutations in humans [10] with inbreeding loops further behind in the pedigree is possible as well as an X-linked disease like NDI caused by *AVPR2* mutations in humans [14]. Even though, both types of mutations usually manifest in the first weeks after birth in humans. As the phenotype in the boars was recognized later in life one can also hypothesize that a dominant mutation in one of these three genes which is located in the upstream, intronic, or downstream regions affecting the expression level is causing the disease. The used approach is only appropriate to detect variants in the coding region and therefore other more comprehensive methods like genome-wide-association mapping in combination with sequencing of the genome of one case could be useful to map the responsible locus in the swine genome and finally to find the causative mutation.

## Conclusions

This case report provides a description of a diagnostic approach to confirm DI in pigs. The report also addresses the limitations and pitfalls while diagnosing DI due to the limited availability of different tests and methods for pigs.

To the author’s knowledge, this report is the first describing DI in Duroc boars. Severe polyuria and polydipsia as major clinical signs indicated an inclusion of Diabetes insipidus in the differential diagnosis. Failure to differentiate polyuric syndromes from other conditions may lead to an incorrect or inconclusive diagnosis of DI. Importantly, a response to ADH administration cannot be used as a diagnostic approach in pigs, because the chemical structure of the product commonly used in humans, cats and dogs is not stimulating the V2 receptors in swine. The observed relationship of the affected animals suggest a possible genetic cause. Although coding mutations in three DI candidate genes can be excluded a genetic background of the disease could not be ruled out and should be carefully investigated in future using more comprehensive methods.
